# The Emergence of Viral Encephalitis in Donkeys by Equid Herpesvirus 8 in China

**DOI:** 10.3389/fmicb.2022.840754

**Published:** 2022-03-03

**Authors:** Tongtong Wang, Leyu Hu, Mengyuan Liu, Tianjiao Wang, Xinyao Hu, Ying Li, Wenqiang Liu, Yubao Li, Yonghui Wang, Huiying Ren, Wei Zhang, Changfa Wang, Liangliang Li

**Affiliations:** ^1^College of Agronomy, Research Institute of Donkey High-Efficiency Breeding and Ecological Feeding, Liaocheng University, Liaocheng, China; ^2^College of Veterinary Medicine, Qingdao Agricultural University, Qingdao, China; ^3^Dairy Cattle Research Center, Shandong Academy of Agricultural Sciences, Jinan, China

**Keywords:** EHV-8, donkey, neurological diseases, pathogenicity, mice

## Abstract

The equine herpesvirus type 8 (EHV-8) can cause significant economic losses in the global horses and donkey industry. The disease has been associated with abortion and respiratory symptoms. However, it is rare for a study to be reported about donkeys with neurological diseases induced by EHV-8 infection. In the present study, one 2-year-old male donkey, from a large-scale donkey farm in China, died with a severe neurological disorder. The causative agent, donkey/Shandong/10/2021 (GenBank accession: OL856098), was identified and isolated from the brain tissue of the dead donkey. Meanwhile, BALB/c mice were used as an animal model to evaluate the pathogenicity of the EHV-8 isolate. Our data showed that EHV-8 was positive in brains by PCR and immunohistochemistry, which induced typical viral encephalitis lesions in both donkey and mice consistent with clinical signs. For the first time, we reported that EHV-8 had been isolated from donkeys with a neurological illness in China, which is helpful to reveal the pathogenicity of EHV-8 in the donkey.

## Introduction

The equine herpesviruses are infectious pathogens that cause serious respiratory diseases and abortion in the equine or donkey industry. Until now, nine herpesviruses have been identified in equids. Horses were considered the natural host to EHV-1–5, while donkeys are for EHV-6–8 (asinine herpesvirus types 1–3, AHV-1–3) (Browning et al., 1988; Azab et al., 2011; Garvey et al., 2018). The natural host of EHV-9 has been unclear until now, although previous studies suggest that some members of the *Equidae* family serve as reservoirs for EHV-9 (Saleh et al., 2017). Notably, EHV-8, EHV-1, EHV-3, EHV-4, and EHV-9 belong to the subfamily *alphaherpesvirinae* (Davison et al., 2009; Liu et al., 2012; Garvey et al., 2018).

EHV-8, a double-stranded enveloped DNA virus 150 kb in length and which contains 76 open reading frames (ORFs) at least (Garvey et al., 2018), has been reported in many countries since it was first isolated from a donkey in Australia in 1987 (Browning et al., 1988). The EHV-8 wh strain was from horses in the northeastern region of China in 2010 (Liu et al., 2012). It has been reported to be present in donkeys in Israel in 2020 (Schvartz et al., 2020). This disease causes serious respiratory disorders and abortion in equine animals. However, the neurological disease induced by EHV-8 in donkeys is limited.

This study described a neurological disorder in donkeys, induced by EHV-8, in China. Furthermore, the pathogenicity of the EHV-8 isolate was assessed in the mouse model. Our data are helpful to improve the understanding of the pathogenicity of EHV-8. These findings extend the spectrum of pathologies potentially attributable to herpesviruses in donkeys.

## Materials and Methods

### Collection of Clinical Samples

In September 2021, five male donkeys with severe neurological symptoms had an outbreak at a large-scale donkey farm in Shandong Province, China. The sick donkeys showed fever, nasal discharge, respiratory signs, anorexia, lethargy, an unsteady gait, and ataxia and were maintained for 2 days, with a case fatality rate of 100%. One of them was submitted to the animal hospital of Liaocheng University for diagnostic testing.

### Pathogen Identification in the Brain

The brain tissue from the dead donkey was collected for bacteriological testing to identify the etiological agent. As previously described, the tissue specimens were cultivated on *Salmonella*–*Shigella* agar and 5% sheep blood agar and incubated at 37°C for 24 h (Wang et al., 2015). Meanwhile, EasyPure^®^ Viral DNA/RNA Kit (Trans Gen Biotech Co., Ltd., China) was used to extract viral RNA and DNA from the brain tissues, according to the instructions of the manufacturer, for virus screening. Seven major pathogens, including EHV-1, EHV-4, EHV-8, H3N8, WNV, JEV, and EAV, were detected by PCR or RT-PCR as per previous reports (Gulati et al., 2012; Wang et al., 2019; Assaid et al., 2020; Yongfeng et al., 2020). All primers used in the present study are listed in Table 1.

**TABLE 1:** The primer sequences in this study.

Primers	Primer sequences (5′–3′)	PCR product sizes
EHV-1 gB -F	GAACCTCAGCCAACCCA	792 bp
EHV-1 gB -R	GCACTTTGCGGACGAAC	
EHV-4 gB -F	CTTAATCGCATTTAGACCGATG	1,591 bp
EHV-4 gB -R	CCGGAACTAGAAAGATGTTATGC	
EHV-8 G1-F	TCAGACTGTCACTCGTGGGA	316 bp
EHV-8 G1-R	CCTGGAGGCCGTTTAACACA	
EAV ORF7-F	ATGGCGTCAAGACGATCAC	333 bp
EAV ORF7-R	TTACGGCCCTGCTGGAGGC	
H3N8 M-F	AAGATGAGTCTTCTGACCGA	1,027 bp
H3N8 M-R	TTACTCCAGCTCTATGTTG AC	
JEV-Prm-F	GGAAATGAAGGCTCAATC	500 bp
JEV-Prm-R	GAAGTCACGATTGCCCATTCC	
WNV-F	GAACGTCAGGTTCCCCCATT	559 bp
WNV-R	GGCGTTGCCGTCATGAAAAT	

### Histopathology and Immunohistochemistry Evaluation

The brain tissues from donkeys or mice were fixed in 10% formalin solution, dehydrated, embedded in paraffin, sectioned to 4-μm thickness, and stained with hematoxylin and eosin for EHV-8 infection detection with positive serum by histopathological analysis and immunohistochemistry (IHC). Finally, the stained tissue samples were observed by light microscopy.

### Virus Isolation and Identification

Virus isolation was attempted on Rabbit Kidney 13 (RK-13) cells as per previous reports (Bazanow et al., 2014). Briefly, the brain tissues mixed with phosphate-buffered saline (PBS) were crushed and homogenized; after that, the tissue homogenate was transferred to a 50-ml sterile centrifuge tube and centrifuged at 12,000 rpm for 15 min at 4°C. The supernatant was collected and filtered through a 0.22-μm syringe filter and then incubated into RK-13 cells. The cytopathogenic effects (CPE) were observed within 3 days following incubation, and the supernatant served as passage 1. After three serial passages, virus isolation was confirmed by PCR with specific primers as detailed above and performed for the next sequencing. Furthermore, indirect immunofluorescence assay was also performed on CPE-positive cells and mock control cells to confirm the EHV-8 isolate. Briefly, these cells were fixed with 75% alcohol; after blocking, these were successively incubated with mouse EHV-8-positive serum that served as primary antibody overnight and with Alexa Fluor 488-labeled Goat Anti-Mouse IgG(H + L) that served as secondary antibody for 1 h at 37°C. After quick staining with 4,6-diamidino-2-phenylindole (Sigma-Aldrich), the samples were analyzed by Leica DMi8 fluorescence microscope, and the images were recorded using Leica X software.

### Growth Curve Design

RK-13 cells were grown to approximately 90% confluency in a petri dish (Nunc, Denmark) and infected with EHV-8 isolate at 0.1 multiplicity of infection. After 1 h of virus adsorption, the cells were washed three times with PBS and incubated in DMEM with 3% FBS at 37°C in a 5% CO_2_ incubator. The supernatant was collected at different time points (12, 24, 36, 48, 60, and 72 h post-infection, hpi), and viral titers were determined in RK-13 cells using the Reed–Muench method.

### Genetic and Phylogenetic Analyses

This study obtained a full sequence of EHV-8 ORF70, and other equid herpesviruses ORF70 sequences (*n* = 26) were downloaded from the GenBank database.^1^ Phylogenetic analysis was completed using MEGA 6.0 software (Masatoshi Nei Lab, State College, United States) by the neighbor-joining method with 1,000 bootstrap replications.

### Mice Model Treatments

Six specific-pathogen-free, 8-week-old male BALB/c mice were obtained from the experimental animal center of Shandong University. The mice were randomly divided into two groups (infected and control) and housed in separate isolators. The mice in the control group were inoculated intranasally with 100 μl MEM, and the infected group was inoculated intranasally with 100 μl EHV-8 solution (including 1 × 10^5^ PFUs). All mice had free access to feed and water and were handled according to appropriate biosecurity guidelines. All experimental protocols were approved by the Liaocheng University Animal Care and Use Committee (permit number: LC2021-10). Post-challenged mice were monitored for 6 days for clinical signs, body weight loss, and mortality. The brain was collected from each mouse to detect EHV-8 by PCR and IHC staining. Meanwhile, histopathological examination was also performed on this tissue.

### The Tracing Investigations

Serum samples were collected randomly from 100 donkeys with no symptoms in the present large-scale donkey farm. The DNA was extracted by EasyPure^®^ Viral DNA/RNA Kit as detailed above. PCR was performed to detect the EHV-8 ORF70 gene in these serum samples using the above-mentioned primers. The cycling conditions were 95°C for 5 min, followed by 35 cycles of denaturation at 95°C for 30 s, annealing at 56°C for 10 s, extension at 72°C for 1 min and 30 s, and final extension at 72°C for 10 min. The productions were confirmed by 1% agarose gel electrophoresis.

### Statistical Analysis

Data are expressed as mean ± standard deviation (SD). Statistical analyses were performed with GraphPad Prism 5 software (GraphPad Software; San Diego, CA, United States). Statistically significant and very significant results were defined by *t*-tests (*P* < 0.05 and *P* < 0.01).

## Results

### Clinical Symptom and Necropsy

**FIGURE 1:**
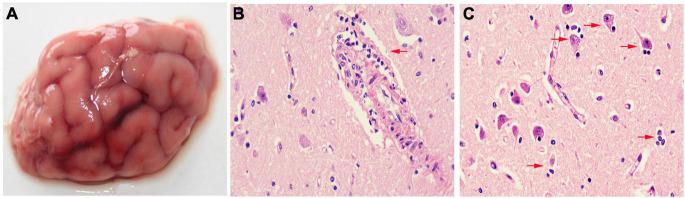
Gross lesions of a dead donkey with a severe neurological disease. **(A)** Moderate meningeal edema lesions in the brain; severe viral encephalitis including perivascular cuffing **(B)**, neuronophagia, and satellitosis **(C)**. HE, ×400.

The donkey showed severe neurological symptoms before death. The necropsy revealed mild meningeal edema lesions in the brain (Figure 1A). The histological examination confirmed that the brain tissue exhibited typical viral encephalitis with perivascular cuffing characterized by lymphocyte infiltration (Figure 1B) and neuronophagia and satellitosis of glial cells characterized by neurons surrounded or phagocytized by gitter cells (Figure 1C).

### Pathogen Identification in the Brain

**FIGURE 2:**
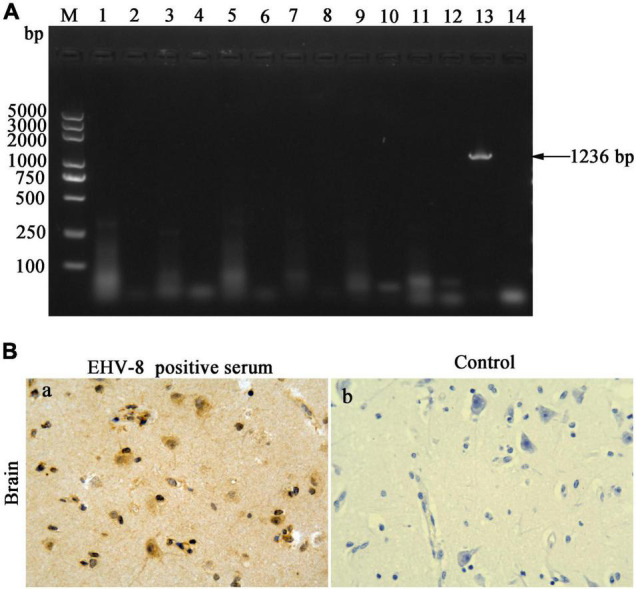
Pathogen identification in the brain. DNA/RNA was extracted from brain tissues; seven pathogens were detected by PCR or RT-PCR **(A)**. Lane M represents a 5,000-bp DNA molecular weight ladder, 1 represents H3N8 detection of brain tissues, 2 represents negative control of H3N8, 3 represents WNV detection of brain tissues, 4 represents negative control of WNV, 5 represents JEV detection of brain tissues, 6 represents negative control of JEV, 7 represents EAV detection of brain tissues, 8 represents negative control of EAV, 9 represents EHV-1 detection of brain tissues, 10 represents negative control of EHV-1, 11 represents EHV-4 detection of brain tissues, 12 represents negative control of EHV-4, 13 represents EHV-8 detection of brain tissues, and 14 represents negative control of EHV-8. **(B)** Immunohistochemical detection of EHV-8 in brain section. Treatment of brain tissue using EHV-8-positive serum as primary antibody **(a)** and negative control brain tissue with mouse IgG substituted for the primary antibody **(b)**. All images are shown at ×40 magnification.

The brain tissues were taken out to uncover potential pathogens. The bacterial culture revealed that no common bacterium was observed on the plates cultivated with the brain tissue of the dead donkey. The PCR/RT-PCR results have proven the brain tissue to be negative for H3N8 (1,027 bp), EAV (333 bp), JEV (500 bp), WNV (559 bp), EHV-1 (792 bp), and EHV-4 (1,591 bp) using specific detecting primers to these viruses but positive for EHV-8, a predicted 1,236-bp (EHV-8 ORF70) product that was identified in the agarose gel (Figure 2A). The brain tissue was sectioned to confirm EHV-8 infection, and viral antigen was detected with immunohistochemistry using EHV-8-positive serum. EHV-8-positive signals appeared brown in neurons due to the presence of diaminobenzidine (Figure 2Ba), but no EHV-8-positive signals were seen in the control section (Figure 2Bb).

### Equine Herpesvirus Type 8 Isolation and Phylogenetic Analysis

**FIGURE 3:**
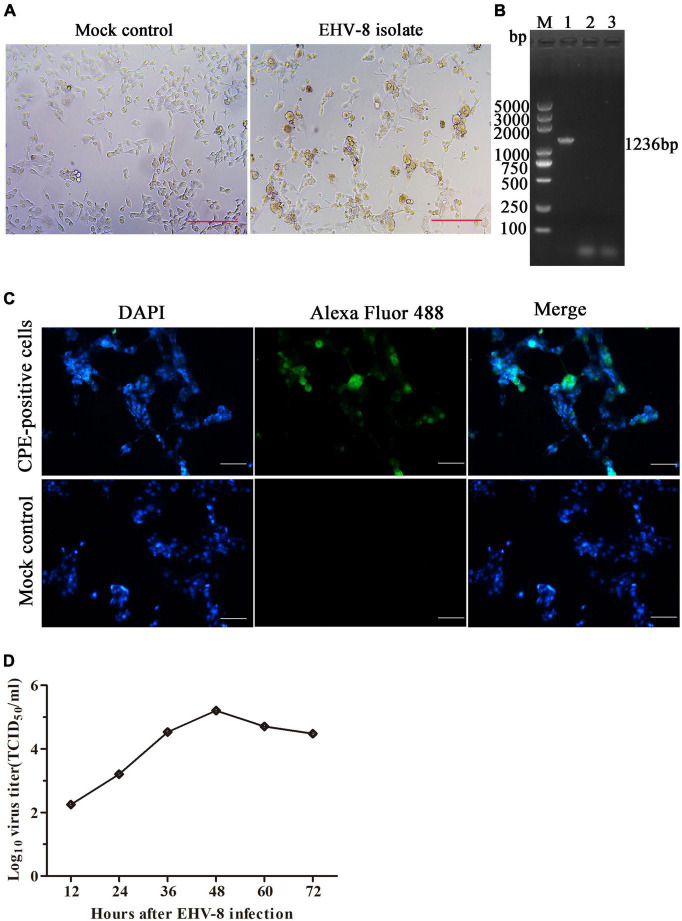
Identification of EHV-8 isolation. The RK-13 cells were inoculated with the supernatant of EHV-8-positive brain samples (right panel) or mock control (left panel). **(A)** The cytopathogenic effect (CPE) was observed using microscopy at 48 h post-infection. Scale bars, 100 μm. **(B)** The ORF70 gene of EHV-8 isolate was determined by PCR detection. The PCR products were analyzed by 1% agarose gel. A marker (lane M) was included on the left, 1 represents CPE-positive cells, 2 represents mock cells, and 3 represents negative control. **(C)** The EHV-8 isolate was further identified by indirect immunofluorescence assay. The images represent the subcellular locations of EHV-8 proteins using indirect immunofluorescence detection with anti-EHV-8 mouse serum and the corresponding Alexa Fluor 488-conjugated secondary antibodies. Cells were imaged by Leica DMi8. Scale bars, 50 μm. **(D)** Growth curve was performed in RK-13 cells to determine the growth characteristics of EHV-8 isolate. Supernatant viruses were harvested at different time intervals in between 12 and 72 hpi, and their titers were determined.

The brain tissue of the dead donkey was homogenized, and the supernatant was inoculated into RK-13 cells for virus isolation. The CPE was observed 3 days post-incubation (Figure 3A). After three serial passages, RK-13 cells were collected to test for EHV-8 using PCR with specific primers (Figure 3B). The CPE-positive cells and mock cells were fixed with 75% alcohol, respectively. Alexa Fluor 488-labeled Goat Anti-Mouse IgG(H + L) served as second antibody. Compared with mock control cells, EHV-8 proteins were observed in the cytoplasm and nucleus of CPE-positive cells (Figure 3C). Furthermore, the *in vitro* proliferation characteristics of the EHV-8 isolate in RK-13 cells were evaluated in a time-course experiment. Figure 3D shows the virus growth pattern in supernatant from 12 to 72 hpi. The maximum virus titer was 5.2 log_10_ TCID_50_/ml on 48 hpi.

**FIGURE 4:**
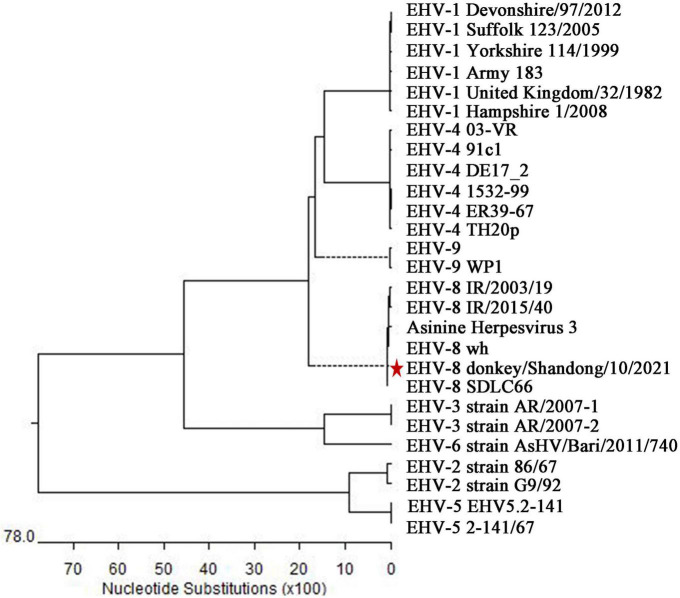
Phylogenetic tree based on the sequence of ORF70 from the isolate in the present study and with known sequences of equine herpesviruses. Genetic variation analysis of isolates detected in the present study based on the phylogenetic trees of the ORF70 gene. The sequences of isolates of ORF70 in the present study are labeled with the scale bar indicates nucleotide substitutions per site. * represent the isolate in the present study.

Finally, the sequence alignment showed that the ORF70 gene sequence of this isolate clustered together and were phylogenetically most closely related to EHV-8, particularly EHV-8 SDLC66, and wh strains, and this sequence was separated from the known sequences of 7 other EHVs (Figure 4) and designated as donkey/Shandong/10/2021 (GenBank accession: OL856098).

### The Pathogenicity of the Equine Herpesvirus Type 8 Isolate in BALB/c Mice

**FIGURE 5:**
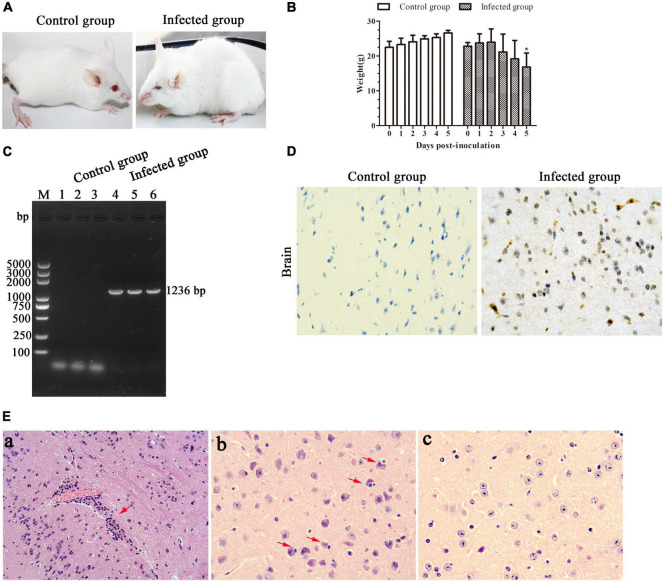
Pathogenicity of the equine herpesvirus type 8 (EHV-8) isolate in mice. Six specific pathogen-free BALB/c mice were randomly divided into the infected and control groups. Clinical signs **(A)** and body weight **(B)** were monitored for 6 days. Differences between groups were assessed using *t*-test, and statistical significances were denoted by *p < 0.05 versus infected group mice at 0 day. The ORF70 gene was detected in brains from the control mice group and infected mice group by PCR **(C)**. Meanwhile, the brain tissues were fixed in 10% formalin solution for immunohistochemistry (IHC) for EHV-8 antigen detection using EHV-8-positive serum **(D)** (IHC × 400) and histopathological analysis **(E)**. **(a)** Perivascular cuffing (HE, ×200); **(b)** neuronophagia of glial cells (HE, ×400); **(c)** mice brain tissue in the control group (IHC, ×400).

In recent years, mice were widely used as an animal model to evaluate EHVs in pathogenicity studies (Mesquita et al., 2017; Abas et al., 2020). In the present study, we found that the BALB/c mice infected with EHV-8 isolate began to show depression, inappetence, ruffled coat, neurological signs, and crouching in corners at 4 dpi (Figure 5A). Meanwhile, the mice had a decreased body weight from 3 dpi and which significantly decreased at 5 dpi (Figure 5B). The appearance of clinical signs in mice was consistent with the reduction of body weight. Subsequently, two mice from the infected group died at 5 dpi (67% mortality). As expected, no obvious clinical signs or significant changes in body weight were observed in the control group.

Furthermore, EHV-8 DNA detection from the brains of BALB/c mice was performed by PCR. The result showed that the EHV-8 ORF70 gene was positive in all brains of the infected mice group and negative in the control group (Figure 5C). Consistent with this, the EHV-8 viral proteins were significant signals in the brain tissue of the infected mice group (Figure 5D). For histopathology, the brains of the infected mice group were induced with typical viral encephalitis lesions. The diagnosis was made when at least one of the following changes was found: perivascular cuffing characterized by lymphocyte infiltration (Figure 5Ea), neuronophagia of glial cells characterized by neurons surrounded by gitter cells (Figure 5Eb), and no significant histopathological changes were observed in the brain tissue of the control mice group (Figure 5Ec).

### The Retrospective and Tracing Investigations of Equine Herpesvirus Type 8

The owners described that several donkeys emerged with neurological disorders at a case fatality rate of 100%. To further determine the prevalence of EHV-8 infection in donkeys, 100 serum samples were collected randomly from the present large-scale donkey farm to detect EHV-8 ORF70 by PCR. The results demonstrated that the positivity rate of EHV-8 was 31% (31/100), indicating that EHV-8 may be one of the most significant causative agents in donkeys.

## Discussion

In the past, viruses such as JEV, H3N8, WNV, and EHV-1 have been predominantly responsible for encephalopathy in horses (Gilkerson and Barrett, 2008; Daly et al., 2011; Burnett, 2016; David and Abraham, 2016; Ali et al., 2020). However, the case of EHV-8 inducing a neurological disease in donkeys has not been reported. In the present study, a severe neurological disorder occurred in one 2-year-old male donkey, the symptoms of which are similar with those of JEV, H3N8, WNV, and EHV-1 induced in horses, as determined through pathogen identification in the brain, ruling out JEV, H3N8, WNV, EAV, EHV-1, and EHV-4 infection. EHV-8 is the sole pathogen recognized by PCR identification, which was isolated on RK-13 cells and named donkey/Shandong/10/2021 strain. This is most closely related to EHV-8, particularly EHV-8 SDLC66 and wh strains associated with respiratory disease and abortion in equids (Liu et al., 2012; Garvey et al., 2018; Schvartz et al., 2020).

In the present case, although we did not culture bacteria from the brain samples of the dead donkey, a histopathological examination of the brain was performed. The details of the microscopic lesions are indicative of typical viral encephalitis, consisting of neuronophagia and satellitosis of neuroglial cells and perivascular cuffing. Meanwhile, IHC was also performed to identify the presence of EHV-8 in neurons. The virus-induced cellular damage to neurons and neuroglial cells might be the reason for the neurological diseases (Cao et al., 2012; Bryant et al., 2018). The dead donkey in the farm was probably the result of the direct action of EHV-8, just like the porcine reproductive and respiratory syndrome virus or pseudorabies virus in pigs (Narita and Ishii, 2004).

Animal models for EHV infection have been specially developed using mice (Azmi and Field, 1993; El-Habashi et al., 2011, 2019; Mesquita et al., 2017). In the present study, the donkey/Shandong/10/2021 EHV-8 can induce a neurological disease in the mice model. The microscopic lesions of mice brains are also similar to the typical viral encephalitis of the donkey. Based on these neuropathological findings and pathogenic features, we speculate that EHV-8 is the primary causative agent inducing a neurological disorder in the donkey. Combined with the 31% EHV-8 positivity rate in the local donkey farm, veterinarians and breeders should be aware of it. More than that, the level of biosecurity and management should be increased in the donkey industry.

## Conclusion

In summary, our study has provided the first evidence of a neurological disease significantly associated with EHV-8 infection in donkeys. The emergence of EHV-8 in donkeys also emphasizes the need for the surveillance of donkey populations in China. Meanwhile, the animal experiment in the mouse model helps enrich the data on EHV-8 pathogenicity.

## Data Availability Statement

The datasets presented in this study can be found in online repositories. The names of the repository/repositories and accession number(s) can be found in the article/supplementary material.

## Ethics Statement

The animal study was reviewed and approved by the Liaocheng University Animal Care and Use Committee. Written informed consent was obtained from the owners for the participation of their animals in this study.

## Author Contributions

TW, LH, and LL conceived, designed the experiments, wrote, and revised the manuscript. ML, TJW, XH, YiL, WL, YuL, YW, HR, and WZ performed the experiments and analyzed the data. CW and LL revised the manuscript. All authors have read and approved the final manuscript.

## Conflict of Interest

The authors declare that the research was conducted in the absence of any commercial or financial relationships that could be construed as a potential conflict of interest.

## Publisher’s Note

All claims expressed in this article are solely those of the authors and do not necessarily represent those of their affiliated organizations, or those of the publisher, the editors and the reviewers. Any product that may be evaluated in this article, or claim that may be made by its manufacturer, is not guaranteed or endorsed by the publisher.

## Footnotes

1
http://www.ncbi.nlm.nih.gov/Genbank
